# Renal microangiopathy induced by lenvatinib in hepatocellular carcinoma: a case report and literature review

**DOI:** 10.3389/fphar.2024.1420377

**Published:** 2024-11-13

**Authors:** Cheng Xue, Linlin Cui, Jiaxin Chen, Yang Liu, Yufei Deng, Wenyi Xu, Zhiguo Mao, Jun Wu

**Affiliations:** ^1^ Department of Nephrology, Shanghai Changzheng Hospital, Second Military Medical University (Naval Medical University), Shanghai, China; ^2^ Department of Biliary Surgery, Eastern Hepatobiliary Surgery Hospital, Naval Medical University, Shanghai, China

**Keywords:** lenvatinib, renal microvascular disease, proteinuria, hepatocellular carcinoma, adverse effects

## Abstract

Lenvatinib, a multi-target inhibitor of receptor tyrosine kinases, has been increasingly used in the treatment of advanced hepatocellular carcinoma (HCC). However, its association with renal adverse effects, including proteinuria and renal microvascular complications, was not fully understood in HCC patients. We reported a case of a 68-year-old male with a history of hypertension, diabetes mellitus, and hepatitis C virus (HCV) infection, diagnosed with primary HCC in 2015. Despite previous treatments, he was started on lenvatinib due to tumor recurrence. Initially, he had mild proteinuria, which significantly worsened post-lenvatinib initiation, accompanied by fluctuating renal function and severe edema. The diagnosis of lenvatinib-induced renal microvascular damage was confirmed through renal biopsy, which showed glomerular sclerosis, tubulointerstitial changes, and arteriolosclerosis. Discontinuation of lenvatinib led to significant improvements in proteinuria and edema. Subsequent cancer recurrence was managed with microwave ablation and immunotherapy, with satisfactory recovery. The potential for lenvatinib to induce significant renal microvascular disease, as demonstrated in this case, emphasizes the importance of vigilant renal monitoring and personalized therapeutic strategies in patients treated with lenvatinib for HCC. Early intervention and dose adjustment may be crucial in preventing severe renal impairment, highlighting the significance of tailored treatment plans in the management of advanced HCC patients especially with pre-existing risk factors.

## 1 Introduction

Hepatocellular carcinoma (HCC) stands as a major health challenge globally, being one of the leading causes of cancer-related mortality. The complexity of HCC, combined with its frequent diagnosis at advanced stages, underscores the critical need for effective systemic therapies. In this context, Lenvatinib has emerged as a noteworthy first-line treatment option.

Lenvatinib, an oral inhibitor targeting multiple tyrosine kinases, has demonstrated significant efficacy in improving clinical outcomes for patients with unresectable HCC. Recent evidence supports the use of Lenvatinib as an effective treatment strategy, either as monotherapy or in combination with other therapeutic modalities such as transarterial chemoembolization (TACE). For instance, a Phase III randomized clinical trial highlighted the enhanced survival benefits of combining Lenvatinib with TACE, presenting a promising first-line treatment for patients with advanced HCC (Peng et al., 2023). Furthermore, Lenvatinib’s non-inferiority to sorafenib, the previous standard of care, has been established, offering an alternative with a favorable safety profile and enhanced efficacy in terms of progression-free survival and objective response rates (Kudo et al., 2018). The therapeutic potential of Lenvatinib extends beyond its direct antitumor effects, with studies suggesting its role in modulating the tumor microenvironment and potentiating immunotherapy responses. This multidimensional therapeutic impact positions Lenvatinib as a cornerstone in the evolving landscape of HCC treatment, offering new avenues for patient management and research.

However, as the utilization of lenvatinib expands in clinical practice, there is a growing awareness of its associated renal adverse events. These events span a spectrum from hypertension and proteinuria to, albeit less commonly, renal failure complications. Such complications underscore the necessity for careful monitoring of renal function and proteinuria in patients undergoing lenvatinib treatment. This vigilance is especially crucial for individuals with existing renal anomalies or risk factors that predispose them to renal impairment. The following case presentation reported the detailed kidney injury of lenvatinib in a recurrent HCC patient, drawing attention to the critical need for timely identification and intervention in managing lenvatinib-induced renal adverse events to prevent irreversible kidney damage.

## 2 Case presentation

**FIGURE 1:**
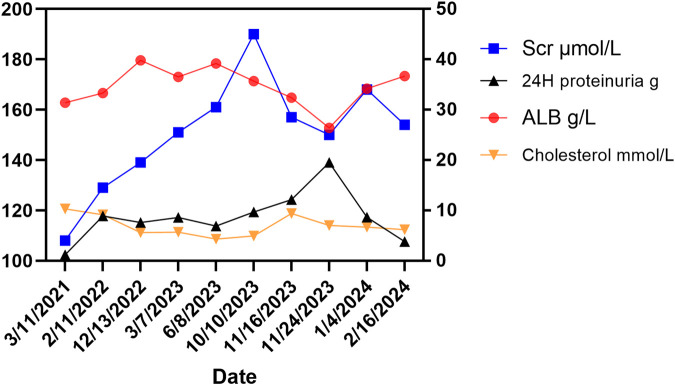
Changes of related indicators before and after using lenvatinib. The right Y-axis showed changes in ALB (albumin, normal range: 35–50 g/L), 24-h proteinuria (normal range: < 0.15 g/d), and cholesterol (normal range: < 5.2 mmol/L) in the patient. The left Y-axis shows the change of serum creatinine (Scr) in the patient (normal range: 60–110 μmol/L for males).

A 68-year-old male, with a history of hypertension, diabetes mellitus, and hepatitis C virus (HCV) infection, was diagnosed with primary HCC in 2015. The timeline was listed in Table 1. Following a series of interventions including partial liver resection, radiofrequency ablation in February 2019, and transarterial chemoembolization in June 2019, he was initiated on a lenvatinib regimen of 12 mg daily in March 2021 after experiencing tumor recurrence. Before the start of this treatment, the patient had a history of mild proteinuria (1+ to 2+) for 5 years, with otherwise normal renal function. Within 10 months of lenvatinib treatment, his urine protein level increased to 3+, and serum creatinine levels rose to 108 μmol/L. Despite reducing the lenvatinib dose to 8 mg daily in October 2022, the patient’s renal function continued to fluctuate, with creatinine levels varying between 120–150 μmol/L and persistent severe proteinuria (3+). By July 2023, he developed significant edema, gaining 10 kg in a month. Despite receiving antihypertensive therapy with irbesartan, nifedipine, and torasemide, his blood pressure remained uncontrolled (160–180/90–110 mmHg). On 24 November 2023, he was admitted to our unit with 4+ proteinuria, a 24-h urine protein excretion of 19.5 g, a creatinine level of 150 μmol/L, hypoalbuminemia (26.4 g/L), and hypercholesterolemia (7.01 mmol/L). Longitudinal evaluations indicated a consistent increase in proteinuria and serum creatinine levels, with notable renal function variability (Figure 1). Testing for PLA2R antibodies was negative.

**TABLE 1:** Summary of the patient’s diagnosis, interventions, and outcomes.

Date/Year	Diagnosis/Intervention	Event/Outcome
2015	Primary HCC diagnosis	Diagnosed with hepatocellular carcinoma (HCC) after hepatitis C infection history
February 2019	Radiofrequency ablation	Tumor ablated successfully
June 2019	Transarterial chemoembolization (TACE)	Additional treatment for tumor recurrence
March 2021	Initiation of lenvatinib (12 mg daily)	Started lenvatinib for recurrent HCC, mild proteinuria present
October 2022	Lenvatinib dose reduced (8 mg daily)	Proteinuria worsened to 3+, creatinine levels fluctuated (120–150 μmol/L)
July 2023	Significant edema, uncontrolled hypertension	Edema and weight gain (10 kg), severe proteinuria (3+), creatinine at 150 μmol/L
November 2023	Hospital admission	Proteinuria (4+), 24-h urine protein 19.5 g, creatinine 150 μmol/L, edema
November 2023	Renal biopsy	Diagnosed with lenvatinib-induced renal microangiopathy and arteriolosclerosis
December 2023	Lenvatinib discontinued	Edema improved, proteinuria reduced to 3+, but renal function remained impaired
January 2024	Microwave ablation, immunotherapy (sintilimab)	HCC recurrence treated successfully, patient discharged after recovery

### 2.1 Biopsy findings

**FIGURE 2:**
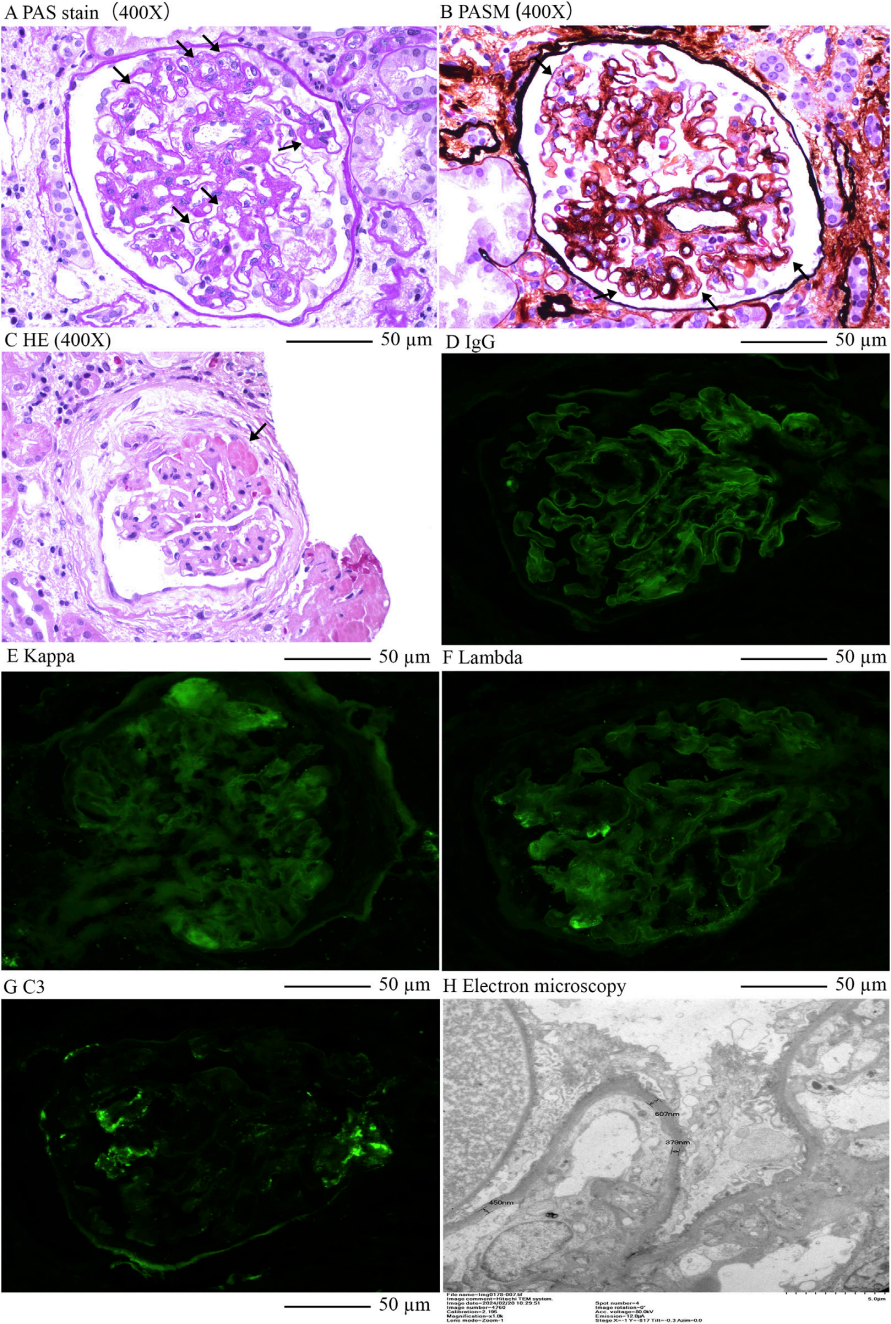
Renal pathology of this case. **(A)**, Enlargement of loose layer on the endothelial side of the glomerular capillary lumen, double contours in capillary loops, Periodic acid–Schiff stain (400X); **(B)**, Swelling and proliferation of endothelial cells in the glomerular capillary lumen; Widening of subendothelial loose layer (Periodic acid–Schiff stain) × 400; **(C)**, Formation of microthrombus in the glomerular capillary lumen, HE×400; **(D)**, Linear stain of IgG in the capillary wall of glomerulus; **(E)**, Kappa light chain staining; **(F)**, Lambda light chain staining; **(G)**, Segmental stain of C3 in the mesengial area of glomerulusL; **(H)**, Electron microscopy indicated double contours in capillary loops and significant local basement membrane thickening.

Renal biopsy revealed microvascular damage (Figure 2). Glomerular observations included 10 globally sclerosed glomeruli and 3 with segmental sclerosis out of 22 assessed, indicating scarring and non-functionality. There was no evidence of acute, severe inflammation. The remaining glomeruli were mildly enlarged, with cell counts of 90–110 per glomerulus and mesangial cells numbering three to five per area, along with mild to moderate mesangial matrix proliferation. This suggested compensatory hypertrophy or hyperfiltration. Capillary loops were poorly patent, some showing “double contour” alterations. The formation of microthrombus was found in the glomerular capillary lumen. Notable periglomerular fibrosis suggested chronic injury. Tubulointerstitial changes included widespread tubular atrophy (60%) and protein casts, alongside diffuse interstitial fibrosis (60%) and inflammation, indicating chronic damage and fibrosis. Vascular pathology showed significant intimal proliferation and hyaline degeneration in small arteries, indicative of arteriolosclerosis, likely related to the patient’s hypertension. Pathological Diagnosis included: 1. Renal Microangiopathy. 2. Renal Arteriolosclerosis.

### 2.2 Management and outcome

Lenvatinib was discontinued, leading to edema improvement with conservative management and diuretics. Proteinuria was reduced to 3+ with a 24-h urine protein excretion of 3.66 g, but renal function remained compromised (creatinine levels at 168 μmol/L). A follow-up liver MRI on 11 January 2024, indicated hepatic cancer recurrence, with an active lesion in the right lobe’s segment VIII. The patient underwent microwave ablation under local anesthesia and ultrasound guidance on 15 January 2024, without complications and was subsequently started on immune therapy with sintilimab, leading to a satisfactory recovery and discharge.

## 3 Discussion

This is a case report about lenvatinib-related renal microvascular disease in HCC. Renal microangiopathy, although rare, emerges as a grave adverse outcome, demanding diligent monitoring and management. This case, augmented by additional literature, casts light on the various manifestations of lenvatinib-induced renal detriment, heralding the critical need for prompt recognition and timely intervention to avert irreversible renal harm in HCC.

In the context of lenvatinib’s renal adverse effects, distinct mechanisms such as severe proteinuria, glomerular damage, or tubulointerstitial nephropathy have been proposed. Therapeutic strategies, including dosage modulation and the employment of oral steroids, have been pinpointed for managing lenvatinib-induced renal failure, accentuating the intricacies of its renal adverse effects and the imperative for customized therapeutic regimens (Cavalieri et al., 2018). Moreover, the incidence of renal failure in patients treated with lenvatinib, as observed in studies like the SELECT trial for thyroid cancer and the REFLECT trial for HCC, underscores the drug’s capacity for renal toxicity. Notably, the SELECT trial reported a 32.2% rate of proteinuria and 4.2% renal failure in patients treated with lenvatinib (Schlumberger et al., 2015), whereas the REFLECT trial observed a 24.8% rate of proteinuria in the context of HCC (Casadei-Gardini et al., 2022). Additionally, renal failure or impairment, including Grade 3 to 5 kidney injury, was reported, with a small percentage of cases resulting in death.

Types of renal damage such as thrombotic microangiopathy, podocytopathy, and renal tubule damage resulting in severe proteinuria and acute renal dysfunction treated with Lenvatinib were mostly reported in patients with thyroid cancer, underscore the drug’s capacity for significant renal impairment (Table 2). Several cases presented biopsy-confirmed renal thrombotic microangiopathy (TMA) in individuals undergoing lenvatinib treatment for thyroid cancer, characterized clinically by proteinuria and stable serum creatinine levels (Hyogo et al., 2018; Contreras Angulo et al., 2023). Moreover, the emergence of focal segmental glomerulosclerosis post-lenvatinib therapy, as observed in a patient with metastatic thyroid cancer, further substantiates the drug’s nephrotoxic potential (Delsante et al., 2022). However, Lenvatinib-induced TMA in HCC is rarely reported. Our case, coupled with the collective insights from various reports, exemplifies the potential for lenvatinib to induce renal local microvascular disease in HCC, which may manifest as a nephrotic syndrome characterized by significant proteinuria and renal dysfunction. These findings advocate for continued alertness to renal impairment signals, such as hypertension, nephrotic syndrome, and acute kidney injury, to facilitate prompt intervention and reduce the risk of chronic renal disease progression (Pham et al., 2022).

**TABLE 2:** Renal injuries and outcome of patients during Lenvatinib therapy.

First author	Year	Gender/Age	Pre-existing conditions	Lenvatinib duration (months)	Kidney diagnosis	Light microscopy	Immunofluorescence	Electron microscopy	Scr (mg/dL)	Therapy	Renal outcome	Follow-up (y)
Pham et al. (2022)	2022	F/68	FTC	22	FSGS TMA Interstitial nephritis	Mesangial expansion Tubular injury and necrosis Interstitial edema and inflammation	NA	GBM and TBM thickening and duplication. Mesangial cell interposition and matrix expansion. Foot process effacement. Endothelial cell swelling with loss of fenestrations. Subendothelial widening	NA	Discontinuation of LEN and treatment with palliative care	Renal function improved and returned to her baseline. Progression of thyroid cancer	<1 dead
Delsante et al. (2022)	2021	M/50	PTC	35	TMA	Arteriolar thrombus Mesangiolysis GBM reduplication	Glomerular vascular pole C3 and fibrinogen	Subendothelial widening. Endothelial swelling. Capillary occlusion	1.1	Discontinuation of LEN	Proteinuria slowly decreased with preserved renal function. Progression of thyroid cancer	<2 dead
Delsante et al. (2022)	2021	F/66	FTC	18	TMA	Mesangiolysis, GBM reduplication	Sparse mesangial IgA	Endothelial swelling. Mesangial cell interposition. GBM deposition	1.0	Discontinuation of LEN	Improvement of proteinuria, Scr remained unchanged, Progression of thyroid cancer	NA
Delsante et al. (2022)	2021	F/53	HTC	8	TMA	Arteriolar onion skinning, Mesangiolysis, GBM reduplication	Negative	NA	0.8	LEN dose was reduced to 10 mg/day	Proteinuria decreased and renal function remained stable, increased thyroglobulin levels	3
Cavalieri et al. (2018)	2018	M/62	PTC	48	TMA FSGS	GBM reduplication Mesangiolysis, Arteriolar necrosis	Negative	FSGS. Foot process effacement. Sparse subendothelial electron deposition	1.9	Discontinuation of LEN	Decreased proteinuria and Scr	1
Furuto et al. (2018)	2018	F/79	PTC	4	FSGS	GBM reduplication FSGS Endothelial swelling	Mild mesangial IgA, IgM, C3, and C4	Foot process effacement. GBM reduplication	1.1	Discontinuation of LEN and use of palliative diuretics	Edema improved and complete remission of proteinuria	1
Fleming et al. (2018)	2018	F/45	MTC	19	FSGS	GBM reduplication FSGS	Negative	Mild GBM reduplication Foot process effacement	NA	Discontinuation of LEN	Decreased Proteinuria	2
Hyogo et al. (2018)	2018	F/70	PTC	56	TMA	GBM reduplication Mesangiolysis	NA	Endothelial swelling and expansion GBM reduplication	1.1	Discontinuation of LEN	Improvement of renal function, decreased proteinuria	<1
Nakashima et al. (2022)	2022	F/77	HCC	2	FSGS TMA	Endothelial swelling GBM reduplication Mesangiolysis Degeneration of the podocytes	Mesangial IgM	Massive subendothelial electron deposition Foot process effacement	1.7	Discontinuation of LEN	Decreased proteinuria, improved kidney function	NA

LEN, lenvatinib; TMA, thrombotic microangiopathies; GBM, glomerular basement membranes; TBM, tubular basement membrane; FSGS, focal segmental glomerulosclerosis; MTC, medullary thyroid cancer; PTC, papillary thyroid carcinoma; HCC, hepatocellular carcinoma; HTC, Hürthle cell thyroid carcinoma; FTC, follicular thyroid carcinoma; eGFR, estimated glomerular filtration rate; NA, not available.

The incidence of renal microangiopathy among patients treated with lenvatinib can be attributed to a complex interplay of factors. On one side, lenvatinib may promote renal vasoconstriction and cause renal damage by the inhibition of VEGF receptors or platelet-derived growth factor receptors (Cavalieri et al., 2018). VEGF plays a critical role in maintaining glomerular capillary integrity and promoting angiogenesis, essential for preserving renal microvasculature. Lenvatinib’s inhibition of VEGF receptors can lead to endothelial cell damage, promoting renal vasoconstriction, microvascular injury, and ultimately, proteinuria and impaired renal function (Cavalieri et al., 2018). Additionally, lenvatinib may induce TMA, which is characterized by the formation of microthrombi in the renal capillaries, further aggravating kidney damage (Cavalieri et al., 2018). On the other side, pre-existing conditions such as renal function, blood pressure control, underlying vascular or inflammatory diseases, etc., can impact the risk of kidney diseases. For example, this patient had a history of hypertension, chronic kidney disease with mild proteinuria, and HCV infection, which may damage the renal filtration barrier and make his kidneys more prone to damage. These conditions may exacerbate the risk of developing severe renal microvascular complications during lenvatinib therapy. A robust baseline health status may offer some degree of protection, indicating the need for comprehensive pre-treatment assessment and ongoing management of comorbid conditions during lenvatinib therapy. Last, the risk of developing renal microangiopathy is influenced by the dosing regimen of lenvatinib. Lower doses or shorter durations of therapy may mitigate the risk of adverse reactions, suggesting that dose adjustments and close monitoring of therapeutic response could be crucial in preventing renal microangiopathy.

Management of lenvatinib-induced renal microangiopathy involves the prompt discontinuation or dose reduction of lenvatinib, along with symptomatic treatment for proteinuria and hypertension. In our case, the cessation of lenvatinib, combined with diuretic therapy and antihypertensive agents, resulted in a significant reduction in proteinuria and edema, although renal function remained compromised. In some cases, corticosteroids or immunosuppressive therapies have been used to manage renal injury, particularly in severe or progressive cases of TMA (Delsante et al., 2022). Regular renal function monitoring, dose adjustment, and early detection of renal impairment are essential in preventing long-term renal damage. It is also critical to evaluate the risks and benefits of continuing lenvatinib therapy in patients who develop renal complications.

Identifying patients at risk for renal microangiopathy before initiating lenvatinib is crucial. Patients with pre-existing conditions such as hypertension, HCV infection, diabetes, or CKD are at a higher risk of developing severe renal complications. Regular monitoring of blood pressure, renal function, and proteinuria during treatment can help detect early signs of renal microangiopathy. Furthermore, the use of renoprotective agents such as angiotensin-converting enzyme inhibitors or angiotensin receptor blockers may offer some protective effects by reducing proteinuria and controlling blood pressure. Early intervention, including dose reduction or discontinuation of lenvatinib at the first sign of renal impairment, can prevent the progression to irreversible renal damage.

From the patient’s perspective, the initial phase of lenvatinib treatment was tolerable, but the worsening proteinuria and severe edema significantly impacted daily life, causing discomfort and limiting mobility. The frequent hospital visits for monitoring added to the burden. After discontinuing lenvatinib, the patient felt relief as the edema improved, and while still concerned about cancer recurrence, they expressed a preference for treatments with fewer renal side effects.

## 4 Conclusion

In conclusion, the multifaceted etiology of lenvatinib-induced renal microvascular disease underlines the criticality of personalized medicine in optimizing lenvatinib utilization in HCC, aiming to balance therapeutic efficacy while minimizing the incidence of severe adverse effects like renal microangiopathy. Therefore, a comprehensive understanding of the diverse renal adverse effects associated with lenvatinib is crucial for clinical decision-making, highlighting the significance of early detection and intervention to mitigate severe renal injury in HCC patients.

## Data Availability

The original contributions presented in the study are included in the article/Supplementary Material, further inquiries can be directed to the corresponding author.
